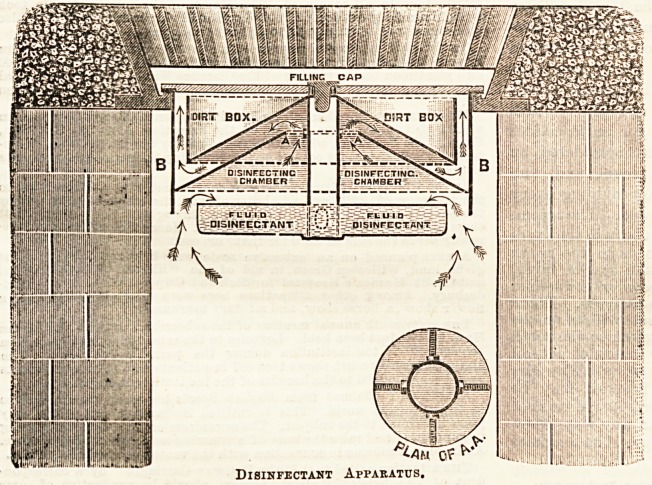# Practical Department

**Published:** 1895-09-14

**Authors:** 


### PRACTICAL DEPARTMENTS.

### The Disposal of Refuse in Birmingham.

A very excellent example is given by the sanitary authorities of the city of Birmingham in the well-systematised way in which the garbage of that great city is disposed of in such a way as least to endanger the public health. Mr. Shaw, in dealing with the subject in his "Municipal Government in Great Britain," gives a full description of the plan adopted in Birmingham. Here an especially designed ash-tub is supplied to each house for the reception of kitchen and other solid refuse, as well as ashes. The contents of these are removed periodically and taken to one of the receiving stations, of which there are several, to all being assigned a position on one of the canal wharves. Here the coarser refuse is committed to furnaces, which number some fifty in all. Of the rest, of some a mixture is made constituting a fertilizer which is promptly removed by means of the canal boats and sold to farmers, while for the most part it is turned into a dry powdered fertilizer in special evaporating machines, worked by the heat generated by the burning of the coarse refuse. Of the latter what remains after the action of the incinerators is a quantity of "clinkers," used for road mending and making, and for concrete and mortar. The extensive canal system of Birmingham naturally is a great aid in the establishment of so complete an organisation by the Health Department. Many boats are possessed by the sanitary authorities for the constant removal of the manufactured fertilizers. Birmingham would seem to have evolved a very satisfactory and well-organised way of dealing with the large quantities of refuse awaiting disposal in a great town, and of turning them to the best advantage compatible with the afety of public health.

### SEWER GAS DESTRUCTOR.

That the present method of disinfecting street drains and sewer openings is more or less insufficient has been demonstrated many a time. The mere distribution of disinfecting powders over the surface of sewer gratings can have very little effect, seeing that the percentage of the actual disinfectant in the best and strongest of these powders is but small, and liable soon to be evaporated, or washed away by rain or other flush of water.

A patent apparatus for the more complete deodorisation and disinfection of noxious gases arising from gulleys and drains has been brought to our notice, the invention of Messrs. I. Miller and Co., sanitary engineers, Commercial Street, E. The apparatus consists of a cylinder of sheet iron (B B), made to fit in the ventilator frame, from a cross-bar in which is suspended a trough, while from the centre rises a tube with holes at the base, having a cap or stopper at the upper end for filling purposes. This trough is filled with a volatile disinfectant. Over it, and suspended from the tube, are fixed two protecting covers, the upper cover reaching from the centre to within one or two inches of the sides of the cylinder, having upright sides forming a dirt box. The lower cover is fixed about an inch below the upper one; its outer

Disinfectant Apparatus.

edges fitting close to the cylinder, but open at the top (A A), so as to allow of the sewer gas passing upwards between the two covers in the direction shown by the arrows. Between the trough and the covers a chamber is thus formed, filled with disinfectant vapour, through which all sewer gas is made to pass, with the intention of thus rendering it harmless before coming into contact with the open air. The apparatus can also be adapted to take powder disinfectants instead of fluids, and to fit any kind or pattern of ventilator grating.

It will be seen from the above description that the result aimed at in this apparatus is to retain an effective disinfectant in position beneath a grating, and to protect it from being washed away by rain or flushings by means of a cover. It is claimed that by this arrangement a saving of labour is effected, as once filled with a sufficiently strong solution it needs no further attention for some time, and the replenishing is easily accomplished by means of the cap at the top of the tube.

Another variety of this apparatus, especially suitable for street gullies, is made in the form of a square box, to fit tightly, with the addition of two flap valves, which are closed except when surface water is passing. Our illustration is given by permission of the patentees.

## Figures and Tables

**Figure f1:**